# Perceptual Characterization of the Macronutrient Picture System (MaPS) for Food Image fMRI

**DOI:** 10.3389/fpsyg.2018.00017

**Published:** 2018-01-26

**Authors:** Jill L. King, S. Nicole Fearnbach, Sreekrishna Ramakrishnapillai, Preetham Shankpal, Paula J. Geiselman, Corby K. Martin, Kori B. Murray, Jason L. Hicks, F. Joseph McClernon, John W. Apolzan, Owen T. Carmichael

**Affiliations:** ^1^Pennington Biomedical Research Center, Baton Rouge, LA, United States; ^2^Department of Psychology, Louisiana State University, Baton Rouge, LA, United States; ^3^Department of Electronics and Communication Engineering, MS Ramaiah University of Applied Sciences, Bangalore, India; ^4^Department of Psychiatry and Behavioral Sciences, Duke University, Durham, NC, United States

**Keywords:** visual paradigm, food preference questionnaire, FPQ, ingestive behavior, reward, BOLD, neuroimaging, eating behavior

## Abstract

Food image fMRI paradigms are used widely for investigating the neural basis of ingestive behavior. However, these paradigms have not been validated in terms of ingestive behavior constructs, engagement of food-relevant neural systems, or test-retest reliability, making the generalizability of study findings unclear. Therefore, we validated the Macronutrient Picture System (MaPS) (McClernon et al., 2013), which includes food images from the six categories represented in the Geiselman Food Preference Questionnaire (FPQ) (Geiselman et al., 1998). Twenty-five healthy young adults (*n* = 21 female, mean age = 20.6 ± 1.1 years, mean BMI = 22.1 ± 1.9 kg/m^2^) rated the MaPS images in terms of visual interest, appetitive quality, nutrition, emotional valence, liking, and frequency of consumption, and completed the FPQ. In a second study, 12 individuals (n=8 female, mean age = 25.0 ± 6.5 years, mean BMI = 28.2 ± 8.7 kg/m^2^) viewed MaPS and control images (vegetables and non-food) during two separate 3T BOLD fMRI scans after fasting overnight. Intuitively, high fat/high sugar (HF/HS) and high fat/high complex carbohydrate (HF/HCCHO) images achieved higher liking and appetitive ratings, and lower nutrition ratings, than low fat/low complex carbohydrate/high protein (LF/LCHO/HP) images on average. Within each food category, FPQ scores correlated strongly with MaPS image liking ratings (*p* < 0.001). Brain activation differences between viewing images of HF/HS and vegetables, and between HF/HCCHO and vegetables, were seen in several reward-related brain regions (e.g., putamen, insula, and medial frontal gyrus). Intra-individual, inter-scan agreement in a summary measure of brain activation differences in seven reward network regions of interest was high (ICC = 0.61), and was even higher when two distinct sets of food images with matching visual ratings were shown in the two scans (ICC = 0.74). These results suggest that the MaPS provides valid representation of food categories and reliably activates food-reward-relevant neural systems.

## Introduction

Food images have been used extensively as stimuli in functional magnetic resonance imaging (fMRI) studies to characterize the neural systems involved in processing the hedonic value of food as well as satiety and hunger signals(Simmons et al., 2005; Beaver et al., 2006; Schur et al., 2009). Food image fMRI studies have provided valuable data about brain functioning among individuals with obesity (Rothemund et al., 2007; Davids et al., 2009), with diagnosed eating disorders (Santel et al., 2006), in a fasting state(Uher et al., 2006; Fuhrer et al., 2008), undergoing modifications to their diet (Simmons et al., 2005; Beaver et al., 2006; Cornier et al., 2007; Schur et al., 2009; Frank et al., 2010), and losing weight (Cornier et al., 2009; Murdaugh et al., 2012). Typically, food image fMRI involves scanning individuals whose hunger and satiety status is known or manipulated, presenting them with photographs of food items and non-food items in the scanner, and asking questions about properties of the shown items (e.g., “How nutritious is this food?”). The fMRI signals are compared between differing experimental conditions; for example, between viewing differing types of foods (high-calorie, sweet, vegetables, etc.), or between fed and fasted states. Relationships between such fMRI signal differences and external variables, such as body mass index, are then evaluated.

Despite widespread use of food image fMRI paradigms, food images have varied broadly from study to study in terms of image content and perceptual properties. Carbohydrate content, fat content, and other nutritional properties differ between food image sets or are not well characterized, leading to potential differences in brain systems engaged by seemingly similar image categories. For example, different high-calorie food image sets may show items such as ice cream and fried chicken, which may differ in hedonic value due to differences in sugar and fat content. Lighting, scale, color, composition, and other perceptual properties can also influence how the brain responds to food images, and these properties differ broadly between available image sets. Attempts have been made to standardize visual properties in available food image sets, but further work is needed to establish the most ecologically valid representation of typically consumed food-related stimuli. The non-food images commonly used as control stimuli show a wide variety of content, including abstract patterns, office furniture, tools, or other items, each of which can elicit different brain responses that impact comparisons to food image responses. Besides influencing how images are perceived and what fMRI signals they evoke, these factors could impact how fMRI signals change over the course of repeated exposure to the images in a longitudinal study, as individuals habituate to the images. In turn, these image-set-specific influences on fMRI signals limit the ability to interpret and generalize the results of food image fMRI studies.

Characterizing the perceptual and psychological properties of food image sets is a key step toward interpreting the results of fMRI studies based on them. Several non-food image sets, including the International Affective Picture System (IAPS) (Libkuman et al., 2007), the Geneva Affective Picture Database (GAPED) (Dan-Glauser and Scherer, 2011), the Bank of Standardized Stimuli (BOSS) (Brodeur et al., 2010), and the set of everyday object drawings developed by Snodgrass and Vanderwart (Snodgrass and Vanderwart, 1980) have been characterized in this way. Alignment between human rater responses and psychological constructs, as well as repeatability and plausibility of evoked brain and behavioral responses, were evaluated. However, to our knowledge, few food-related image sets have been characterized in this way, using either subjective participant ratings or fMRI data (Blechert et al., 2014; Charbonnier et al., 2016). While we know that high-calorie food images are typically rated higher in liking and palatability and evoke increased reward-related brain activity compared to low-calorie images (Killgore and Yurgelun-Todd, 2005; Stoeckel et al., 2008; Siep et al., 2009; Dimitropoulos et al., 2012; Murdaugh et al., 2012; Ohla et al., 2012), we are still unsure whether these differences are driven purely by energy density, or by the macronutrient composition of the foods. Establishing a set of standardized macronutrient cutoffs that can be applied to existing and future food image sets will allow us to better disentangle this question.

The aim of this study was to characterize and validate one food image set, the Macronutrient Picture System (MaPS) (McClernon et al., 2013), in terms of its relationship to established ingestive behavior constructs and the plausibility and repeatability of its evoked fMRI signals. The MaPS is based on the same experimental design as the Geiselman Food Preference Questionnaire (FPQ) (Geiselman et al., 1998), a rigorously validated survey that determines an individual's food preferences by having participants rate their liking of foods in the six food categories detailed the *Materials* section below. Because the MaPS was built from the same paradigm as the FPQ, it was expected that participant responses on the FPQ would correlate strongly with responses to the MaPS images. MaPS images of high fat, high carbohydrate foods were also expected to elicit robust, repeatable brain activation patterns similar to those identified in previous appetite and reward related fMRI work using high-calorie or high-energy-dense foods. The chief goal of these studies was to establish if these predictions were supported, and therefore whether the MaPS can be considered a valid portrayal of foods in the six food categories. The study provides a template for food image set characterization that can be followed by other groups, leading to a principled basis of comparison among food image sets and the fMRI studies based on them.

## Methods

### Study 1: behavioral study

#### Participants

Twenty-five undergraduate students enrolled in psychology courses at Louisiana State University provided ratings of MaPS images. All participants had normal or corrected to normal vision by self-report, no participants had any dietary restrictions (i.e., vegetarian, vegan) or history of eating disorders, and they could not be on medications that must be taken with food due to the fasting requirement. All participants began experimental procedures at approximately 10:30 a.m. following an overnight fast that was instructed to begin at 10:00 p.m. the night prior. Students were recruited via an online system that provides psychology course credit for research participation. The Institutional Review Board of The Louisiana State University approved this study and informed consent was obtained from every individual prior to their involvement in the study.

#### Materials

##### Geiselman food preference questionnaire

Participants completed the FPQ (Geiselman et al., 1998), which asks participants how much they like food items from six different categories. There are three carbohydrate categories: high simple sugar (HS), high complex carbohydrate (HCCHO), and low carbohydrate/high protein (LCHO/HP). Each of the three carbohydrate categories is subdivided by fat content into high fat (HF) and low fat (LF), resulting in six total food categories: high fat/high simple sugar (HF/HS), low fat/high simple sugar (LF/HS), high fat/high complex carbohydrate (HF/HCCHO), low fat/high complex carbohydrate (LF/HCCHO), high fat/low carbohydrate/high protein (HF/LCHO/HP), and low fat/low carbohydrate/high protein (LF/LCHO/HP). Each food item in the three high-fat cells is greater than 45% fat (expressed as percent of the total kilocalories in a given food), and each food in the three low-fat cells is less than 20% fat. Foods in each of the two high-sugar cells are greater than 30% sugar, and foods in each of the two high complex carbohydrate cells are greater than 30% complex carbohydrate. Foods in each of the two high-protein cells are greater than 13% protein; however, most foods in these two cells are 20–35% protein. The FPQ contains a total of 72 foods with 12 foods in each of the six cells, listed in random order. Participants rated each food item listed (only a written list, no food images) for liking on a scale of one to nine; one being “Dislike Extremely” and nine being “Like Extremely.” Only whole numbers could be chosen. These ratings were averaged to determine a liking score for each of the six food categories for each participant (Geiselman et al., 1998).

##### MaPS

Participants viewed the MaPS which consists of 144 images from six different categories (MaPS version 1.0; 2013). These categories vary in fat, sugar, complex carbohydrate, and protein content, following the experimental design used in the Geiselman FPQ and the Geiselman Macronutrient Self-Selection Paradigm (MSSP) (Geiselman et al., 1998). Twenty-four images from each category were selected from web image searches of specific foods. There was double representation of each of the 72 food items from the FPQ, resulting in an overall set of 144 images. Pictures were acquired by the researchers when web images were not available. Example food items from each category are listed in Table 1. Example images are shown in Appendix A (Supplementary Material).

**Table 1:** Example foods in each category of the Macronutrient Picture System (MaPS) and the Geiselman Food Preference Questionnaire (FPQ).

	**High simple sugar (HS)**	**High complex carbohydrate (HCCHO)**	**Low carbohydrate/high protein (LCHO/HP)**
High Fat (HF)	Cakes	French fries	Steak
	Cookies	Potato chips	Eggs
	Candy bars	Cheese pizza	Fried chicken
Low Fat (LF)	Fresh fruit	Plain rice	Baked chicken
	Gummy candy	Wheat bagels	Green vegetables
	Hard candy	Corn	Broiled fish

##### MaPS rating questions

Participants rated the 144 images on appetizing quality, perceived nutrition, and liking to determine if these properties differed across carbohydrate factor or fat content as expected (Goldstone et al., 2009). Ratings were also collected on visual interest, emotional valence (Cardello et al., 2012), and frequency of consumption (Holley et al., 2015), as these factors could influence appetizing quality, perceived nutrition, and liking ratings. Each rating was provided on a visual analog scale (VAS) with a zero to 10 range, with zero always being the least and 10 always being the greatest. VAS ratings such as these have been shown to reliably represent participant assessment of appetitive sensations (Flint et al., 2000). Each participant was seated at a computer work station and shown the food images on the computer screen using Windows Picture Viewer. Images were displayed one at a time and viewed for the amount of time it took to verbally rate each image on the six criteria described previously. Images were presented in a fixed category order (i.e., HF/HS then LF/HS then HF/HCCHO then LF/HCCHO then HF/LCHO/HP then LF/LCHO/HP then HF/HS again), but the first category seen varied across participants. The proctor of the experimental session did not view the images while the participants saw them, to reduce proctor influences on ratings. After rating the images, participants provided demographic information (age, gender, ethnicity, etc.), information about hunger status currently and during the previous week (VAS rating scale of 0–10 from “Not Hungry at All” to “The Most Hungry I Have Ever Been”), and liking ratings on the FPQ (Geiselman et al., 1998). Finally, height and weight were measured. The session took approximately 2 h.

#### Data analysis

Means and standard deviations of participant ratings were calculated within each image category. A repeated measures 3 × 2 ANOVA was conducted with carbohydrate factor (HS, HCCHO, and LCHO/HP) as one variable, and fat factor (HF and LF), as the other variable. This ANOVA was conducted six times, once for each rating question. *Post-hoc* paired *t*-tests were conducted to determine pairwise differences among the three levels of the carbohydrate factor.

Within each image category Shapiro-Wilk tests of normality were examined to assess whether the ratings were normally distributed, and z-scores for each rating were calculated to identify images with outlier ratings. Within each image category, a summary FPQ liking score for each participant was calculated by averaging the FPQ response to every food in that category, and this FPQ liking score was related to the corresponding mean image liking rating for that category through linear regression and ANOVA. All statistics were calculated using SPSS 22.0 or Microsoft Excel 2010.

### Study 2: fMRI study

#### Participants

Twelve individuals were recruited via the LSU psychology course credit system, via word of mouth, and via the Pennington Biomedical Research Center web site for fMRI scanning during MaPS photograph viewing. All participants had normal or corrected to normal vision by self-report, no participants had any dietary restrictions (i.e., vegetarian, vegan) or history of eating disorders, and they could not be on medications that must be taken with food due to the fasting requirement. All participants received fMRI scans prior to 12:30 p.m. the day following an overnight fast that was instructed to begin 10:00 p.m. the night prior. Most scans occurred prior to 9:00 a.m. Students received psychology course credit through the online system, while non-students received a $50 gift card for participation. The Institutional Review Board of the Pennington Biomedical Research Center approved this study. Informed consent was obtained from every individual prior to their involvement in the study.

### MRI data acquisition

Participants in the fMRI study received MRI scans on a GE Discovery 750w 3.0T scanner with a 32-channel head coil. Participants wore a respiratory monitoring belt and pulse oxygenation sensor during scanning to allow *post-hoc* correction of cardiac and respiratory influences on fMRI using the RETROICOR algorithm (Glover et al., 2000). EPI BOLD fMRI data was collected with the following parameters: Voxel size: 3 × 3 × 3 mm, 96 × 96 × 43 voxels, TR: 3,000 ms, TE: 35 ms, flip angle: 90, bandwidth: 250, NEX: 1, single shot. Participants also received a T1-weighted FSPGR BRAVO structural acquisition for anatomical reference. Key parameters include: voxel size: 0.94 × 0.94 × 1.2 mm, 256 × 256 × 140 voxels, TR:8.5, TE:3.3, TI:450 ms, flip angle:12, bandwidth:31.25, NEX:2, Time: 3:22.

### fMRI task design

During the fMRI task, blocks of HF/HS and HF/HCCHO images from the MaPS, along with blocks of vegetables and non-food control images, were projected onto a bore-mounted screen and viewed through a head-coil-mounted mirror. The two categories of MaPS image types were selected because they represent foods that tend to have higher caloric values (HF/HS, HF/HCCHO) in the image set. A separate set of non-MaPS vegetable images and non-food control images were used for comparison against the HF/HS and HF/HCCHO. Vegetable images were sourced similarly to MaPS. Non-food control images used were MaPS images that had been blurred beyond recognition. See Appendix A (Supplementary Material) for example images. Images were presented in a randomized block design of ten images from a single category per block. Each image category was shown twice, for a total of eight blocks in a single run paradigm. Each image was presented for 5 s. To encourage continued participant engagement in the task, after each image presentation participants were prompted by the onscreen question, “Do you like to eat this?” Participants responded “yes” or “no” via button boxes. Participants had 5 s to respond before the next image automatically displayed. A rest period randomly timed between 30 and 50 s occurred between blocks. Each scanning session took approximately 40 min.

Each participant completed two MRI scans to assess inter-scan repeatability of fMRI signals **(**inter-scan interval: range of 2–30 days, average 9.21 ± 8.96 days). The participants were split into two groups to explore differing strategies for ensuring repeatability of fMRI data while maintaining alertness and engagement in the task. Specifically, participants were randomized into a “repeated image set” group (*n* = 6; mean age 28.5 ± 7.9 years; mean BMI 32.56 ± 8.66 kg/m^2^), or a “novel image set” group (*n* = 6; mean age 20.16 ± 4.19 years; mean BMI 22.56 ± 3.9 kg/m^2^). Participants in the repeated image set group viewed the same set of HF/HS and HF/HCCHO MaPS images at both sessions. Participants in the novel image set group viewed different sets of HF/HS and HF/HCCHO images during their first and second scans. The two sets of HF/HS images were constructed by randomly splitting the full set of HF/HS images in half, and swapping images between subsets to achieve highly similar subjective image ratings between the subsets. The two sets of HF/HCCHO images were constructed similarly. Vegetable images and non-food control images were the same for all participants at every scan session. The average length of functional scanning run was 14.2 min.

#### Data analysis

##### fMRI data preprocessing

Preprocessing of fMRI data used Statistical Parametric Mapping 12 (SPM12). Preprocessing included slice timing correction, head motion correction, smoothing, co-registration to the T1-weighted scan, and warping the T1-weighted data and thus fMRI data to a standard coordinate frame (Montreal Neurological Institute, MNI). Cardiac and respiratory components of the time series were removed using the RETROICOR algorithm (Holley et al., 2015). Time points exhibiting excess head motion (defined as greater than 1.5 degrees of rotation or 1.5 mm of translation) were identified and removed from the analysis. Each participant's data for each condition was entered into a first-level voxel-wise analysis using the General Linear Model. A boxcar function is modeled inside each image block and convolved with the canonical hemodynamic response function.

##### fMRI activation analysis

First-level beta maps quantified, at an individual level, differences in the BOLD signal according to four contrasts: HF/HS vs. vegetables; HF/HCCHO vs. vegetables; HF/HS vs. control; HF/HCCHO vs. control. Pre-selected ROIs [putamen, superior temporal gyrus, medial frontal gyrus, cingulate gyrus, parahippocampal gyrus, insula, orbitofrontal cortex, ventral striatum; Appendix C (Supplementary Material)] were of primary interest based on a previous meta-analysis of reward network fMRI activation in humans (Coletta et al., 2009). These ROIs were used to provide univariate summaries of beta values of activated or deactivated voxels for the four contrasts of interest. Voxels within these ROIs were identified by transforming the Automated Anatomical Labeling (AAL) and AAL2 atlases, which contain labels for each of these regions, into MNI space. The ventral striatum ROIs were defined separately as spheres with a 5 mm radius with center coordinates x = ±18, y = 12, z = −6 (Jensen et al., 2003). For all contrasts of interest, voxels with beta values above a pre-defined positive threshold (+0.2) were extracted across each ROI and defined to be activated voxels; those with beta below a pre-defined negative threshold (−0.2) were extracted across each ROI and defined to be the deactivated voxels.

Mean beta value among activated voxels was our ROI-level fMRI activation summary and primary fMRI outcome of interest. The repeatability of this univariate activation summary across imaging sessions was assessed using the one-way analysis of variance form of intra-class correlation coefficient, ICC (3,1) (Shrout and Fleiss, 1979).

A voxel-based analysis then identified any BOLD signal changes that, at a group level, differed between image categories but fell outside of our prescribed ROIs. The individual-level contrast maps described above were entered into second-level analyses in SPM12 for this purpose. The inter-scan interval in terms of number of days between the two sessions and the BMI of each participant were included as covariates during the second level analysis. These second level analyses resulted in *P*-value maps that were corrected for multiple comparisons using a false discovery rate (FDR) of *p* < 0.05. We assessed whether the ROIs implicated in this voxel-based analysis are biologically plausible considering what is known in the literature about food image-related brain activation.

## Results

### Participants

Participants in the behavioral study (*n* = 4 male, *n* = 21 female) had a mean age of 20.6 ± 1.1 years and a mean BMI of 22.1 ± 1.9 kg/m^2^. Participants reported an average current hunger score of 7.48 ± 1.50 and an average hunger over the previous week score of 5.20 ± 1.71 on the VAS rating scale (0–10 anchored with “Not Hungry at All” to “The Most Hungry I Have Ever Been”). A separate sample of four males and eight females were recruited into the fMRI study, with a mean age of 25.0 ± 6.5 years and a mean BMI of 28.2 ± 8.7 kg/m^2^.

### Study 1: behavioral study

#### Distribution of image ratings

Normality tests indicated a normal distribution of image ratings in each category for each image. There was no significant effect of race (64% Caucasian, 16% African American, 12% Asian, 4% Hispanic). The highly skewed sex distribution precluded exploration of sex differences in ratings. See Appendix B (Supplementary Material) for average image ratings data.

#### Differences in image rating by macronutrient category

For the purposes of these analyses, results are presented for the fat categories (HF vs. LF) collapsed across carbohydrates, and for the carbohydrate categories (HS vs. HCCHO vs. LCHO/HP) collapsed across fats. HF images elicited higher interest than LF images [*F*_(1, 24)_ = 11.80, *p* = 0.002]. HS images were rated as significantly more interesting than HCCHO and LCHO/HP [*F*_(2, 24)_ = 4.99, *p* = 0.011, *post-hoc t*_(24)_ = 2.96, *p* = 0.007 and *t*_(24)_ = 2.28, *p* = 0.031]. HF images were rated as more appetitive [*F*_(1, 24)_ = 14.01, *p* = 0.001] and less nutritious [*F*_(1, 24)_ = 442.32, *p* < 0.0001] than LF images. LCHO/HP were rated as more nutritious than HCCHO and HS [*F*_(2, 24)_ = 119.88, *p* < 0.0001, *post-hoc t*_(24)_ = −32.04, *p* < 0.0001 and *t*_(24)_ = −26.54, *p* < 0.0001], and HCCHO was rated more nutritious than HS [*t*_(24)_ = −3.56, *p* = 0.002]. HF images elicited higher emotion ratings than LF images [*F*_(2, 24)_ = 8.46, *p* = 0.008]. HF images were better liked than LF images [*F*_(2, 24)_ = 7.57, *p* = 0.011]. Frequency of consumption ratings differed by carbohydrate content [*F*_(2, 24)_ = 19.92, *p* < 0.0001], with HS foods consumed less frequently than HCCHO [*t*_(24)_ = −8.83, *p* < 0.0001] and LCHO/HP [*t*_(24)_ = −5.30, *p* < 0.0001]. No significant difference between HCCHO and LCHO/HP consumption frequency was seen. These results are summarized in Tables 2, 3.

**Table 2:** Means and standard deviation of image ratings collapsed into high fat and low fat categories.

	**High fat (HF)**	**Low fat (LF)**
Interest	5.25 ± 1.05^*^	4.56 ± 1.35
Appetite	5.74 ± 1.40^*^	4.73 ± 1.45
Nutrition	2.28 ± 0.75^*^	5.27 ± 0.75
Emotion	3.61 ± 1.70^*^	3.17 ± 1.70
Liking	5.57 ± 1.20^*^	4.90 ± 1.10
Frequency	3.14 ± 0.90	3.21 ± 1.00

* *Significantly different from Low Fat category (p < 0.05)*.

**Table 3:** Means and standard deviation of image ratings collapsed into carbohydrate categories.

	**High sugar (HS)**	**High complex carbohydrate (HCCHO)**	**Low carbohydrate/high protein (LCHO/HP)**
Interest	5.39 ± 1.45^b^	4.67 ± 1.40^a^	4.65 ± 1.10^a^
Appetite	5.20 ± 1.50	5.51 ± 1.30	5.01 ± 1.60
Nutrition	2.65 ± 0.45^a^	3.01 ± 0.85^b^	5.67 ± 1.20^c^
Emotion	3.64 ± 1.85	3.26 ± 1.70	3.27 ± 1.75
Liking	5.10 ± 1.30	5.54 ± 1.20	5.06 ± 1.30
Frequency	2.56 ± 0.75^b^	3.46 ± 0.90^a^	3.50 ± 1.10^a^

*Values with different superscripts are significantly different at p < 0.05*.

#### Relationship between image rating and FPQ score

**Figure 1:**
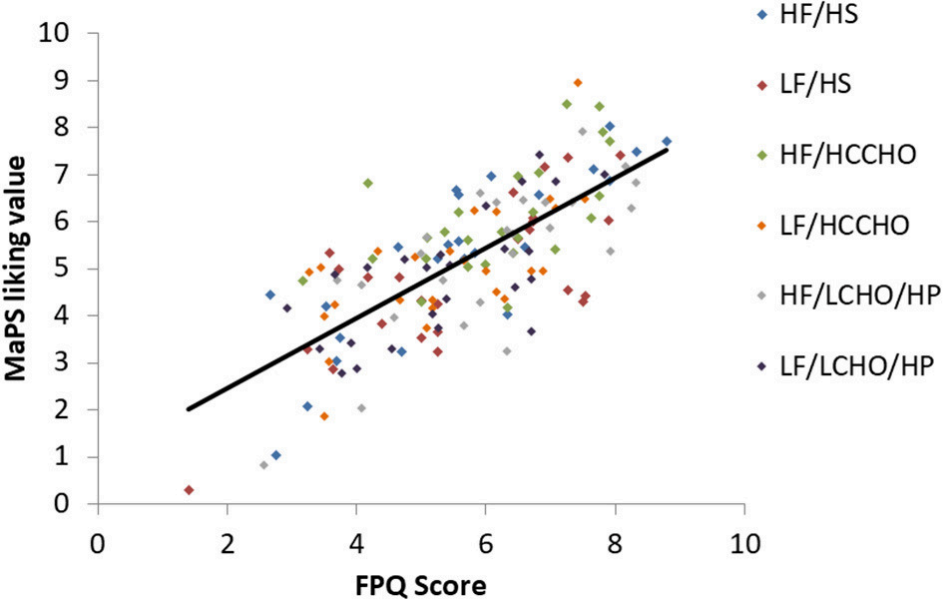
Scatterplot depicting the relationship between FPQ and MaPS liking scores by image category. Overall trendline *R*^2^ = 0.5422, *p* < 0.0001.

Results are presented for each of the six food image categories. Greater FPQ scores were associated with greater MaPS image liking ratings overall [*F*_(1, 148)_ = 175.29, *p* < 0.0001, *R*^2^ = 0.5422, Figure 1] and within each category: HF/HS [*F*_(1, 23)_ = 58.68, *p* < 0.0001, *R*^2^ = 0.7184], LF/HS [*F*_(1, 23)_ = 26.35, *p* < 0.0001, *R*^2^ = 0.5339], HF/HCCHO [*F*_(1, 23)_ = 12.66, *p* = 0.002, *R*^2^ = 0.3551], LF/HCCHO [*F*_(1, 23)_ = 18.69, *p* = 0.0002, *R*^2^ = 0.4483], HF/LCHO/HP [*F*_(1, 23)_ = 30.66, *p* < 0.0001, *R*^2^ = 0.5713], and LF/LCHO/HP [*F*_(1, 23)_ = 20.82, *p* = 0.0001, *R*^2^ = 0.4751; see Figure 1 and Appendix B (Supplementary Material)].

### Study 2: fMRI study

#### Repeatability

The ROI-level activation summary was reliable across scan sessions (ICC: 0.74 for novel image set and 0.61 for repeated image set). Note that these ICC values are competitive with those of prior fMRI studies(Caceres et al., 2009; Fliessbach et al., 2010; Sheu et al., 2012; Brandt et al., 2013; Somandepalli et al., 2015). Linear regression between first and second session activations suggested that in the repeated image set group, ROI activations tended to be similar, but smaller in magnitude, in the second session (linear regression slope: 0.93), while activation magnitudes were more similar between sessions within the novel image set group (slope: 1.03). This suggests that the lower ICC in the repeated image set group may at least partially reflect a practice effect that reduced activation magnitude in the second session.

#### Activated regions

**Figure 2:**
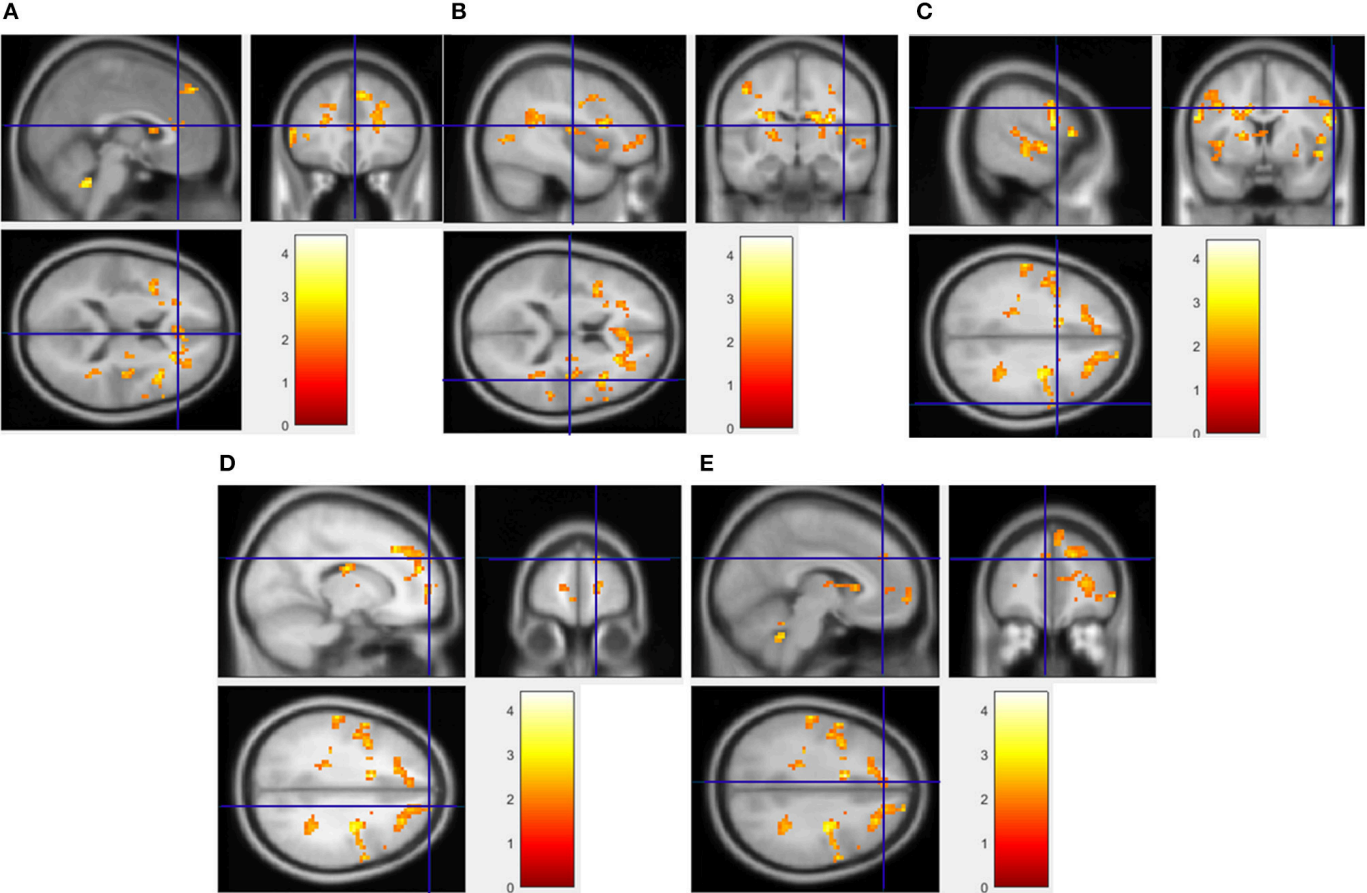
Regions showing significantly greater activation to HF/HS images compared to vegetables. Implicated regions (shown with blue cross-hairs) include the anterior cingulate gyrus **(A)**, insula **(B)**, and superior frontal **(C)**, precentral **(D)**, and medial frontal gyrus **(E)**. Activation differences between HF/HCCHO and vegetables are similar (not shown).

**Figure 3:**
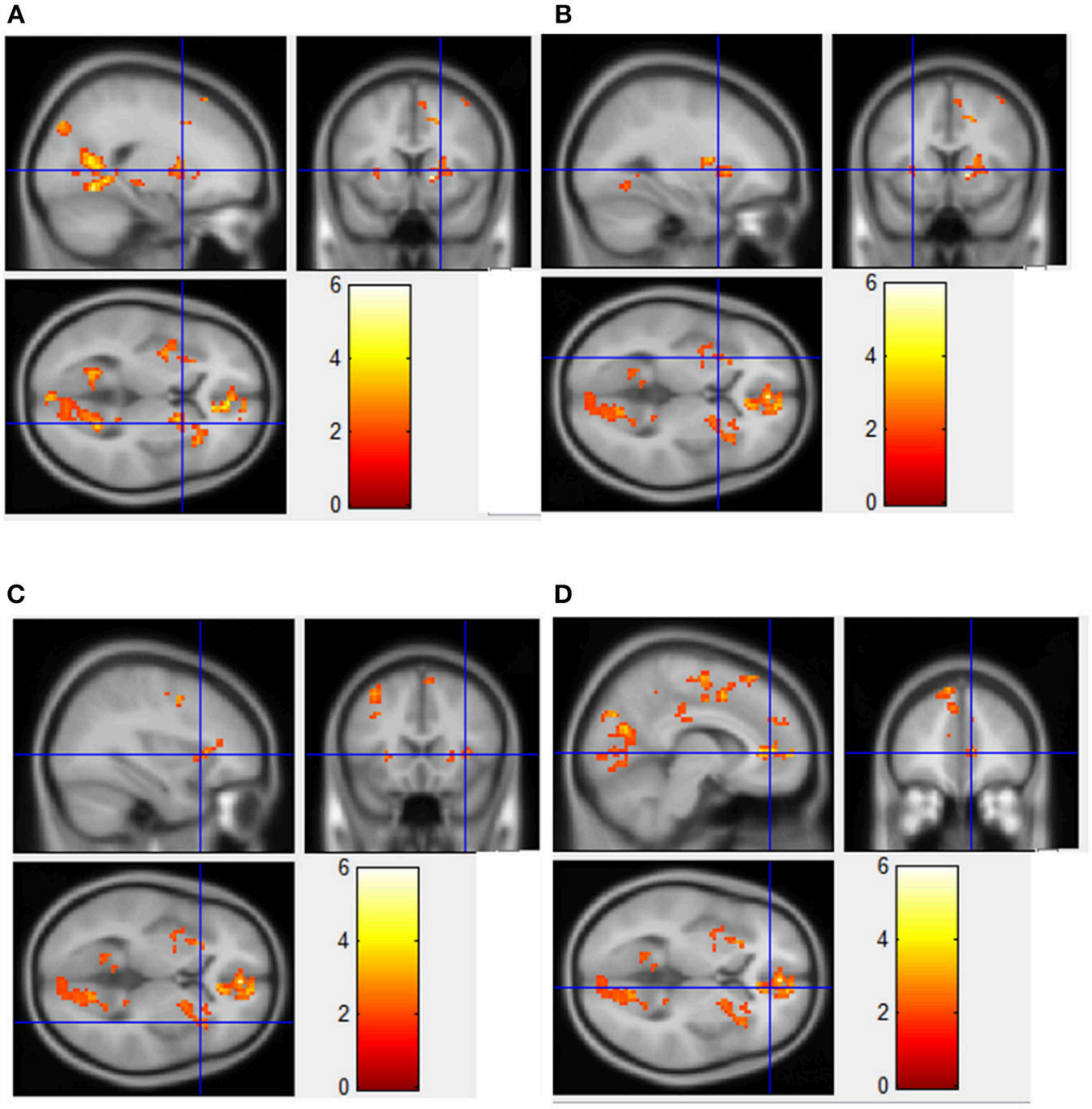
Regions showing significantly greater fMRI activation during viewing of HF/HCCHO images compared to non-food control images. Implicated regions (shown with blue cross-hairs) include the right putamen **(A)**; left putamen **(B)**; right insula **(C)**; anterior cingulate cortex **(D)**.

**Figure 4:**
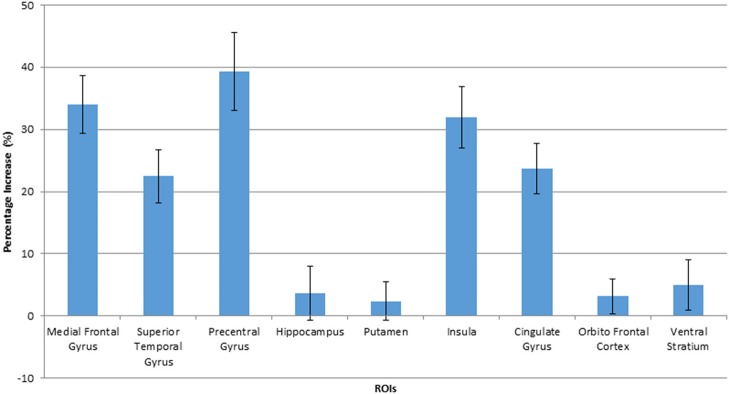
Percentage increase in BOLD signals when viewing HF/HCCHO images, compared to vegetables, in activated ROIs. Values are expressed as a percentage of the BOLD signal response to vegetable images. The error bars show the standard deviation.

The voxel-level analysis identified significant clusters of brain activation in several of the pre-selected ROIs mentioned above. The anterior cingulate cortex, insula, cingulate gyrus, medial frontal gyrus, precentral gyrus and the superior frontal gyrus displayed significant activation, but putamen, parahippocampal gyrus, orbitofrontal cortex and ventral striatum did not, during HF/FS image viewing compared to vegetables [Figure 2, voxel *p*-value (FDR) = 0.05, voxel extent = 20]. This finding agrees with prior reports (Simmons et al., 2005; Coletta et al., 2009). Highly similar patterns of activation were seen in response to HF/HCCHO images, compared to vegetables. Specifically, the insula, anterior cingulate cortex, cingulate gyrus, medial frontal gyrus, and precentral gyrus (all *p* < 0.05) were activated to a greater extent for HF/HCCHO images compared to vegetables. Activations were also visible in regions of the visual cortex in confirmatory whole brain analyses (data not shown). HF/HCCHO images evoked significantly greater activation than non-food control images in a more restricted set of structures, especially the insula, putamen and also the anterior cingulate cortex (all *p* < 0.05) (Figure 3); corresponding differences between HF/HS and non-food control images showed a similar pattern, including the insula and anterior cingulate cortex (both *p* < 0.05) (data not shown). Among regions with significant differences in activation between HF/HS vs. vegetables and HF/HCCHO vs. vegetables, the magnitude of the difference was greatest in cortical regions (i.e., precentral, cingulate, superior temporal, and medial frontal gyri), and least in subcortical structures (i.e., putamen and hippocampus, see Figure 4). Activation analyses were carried out both with and without BMI and inter-scan interval as covariates. In both cases activations were observed in the above mentioned areas for *p* < 0.05. These results with covariates are summarized in Table 4. In confirmatory whole brain analyses, significant activations outside of the reward network of ROIs were observed primarily in the visual cortex (*p* < 0.05).

**Table 4:** Regions showing brain activations in response to viewing food photos. Coordinates of regions are shown in Appendix C (Supplementary Material).

	**Region**	**Cluster size**	***P* < 0.05 FDR corrected**
HF/FS vs. Vegetables	Anterior cingulate cortex	28	0.05
	Insula	24	0.05
	Cingulate gyrus	31	0.03
	Medial frontal gyrus	26	0.05
	Precentral gyrus	21	0.05
	Superior frontal gyrus	27	0.05
HF/HCCHO vs. Vegetables	Insula	43	0.03
	Anterior cingulate cortex	26	0.04
	Cingulate gyrus	22	0.05
	Medial frontal gyrus	34	0.05
	Precentral gyrus	28	0.05
HF/HCCHO vs. non-food	Insula	33	0.03
	Putamen	52	0.05
	Anterior cingulate cortex	31	0.04
HF/HS vs. non-food control	Insula	26	0.05
	Anterior cingulate cortex	29	0.03

## Discussion

The key finding of this study is that the MaPS elicits valid behavioral responses to food images, as well as valid and repeatable brain responses to food images. The MaPS is thus a promising tool for valid and reliable food image fMRI paradigms. According to participant ratings, the image set probes known ingestive behavior constructs in expected ways: high fat foods are more well-liked and appetizing, while low fat foods are perceived as more nutritious. The significant positive correlation between the MaPS and the FPQ, an already validated measure (Geiselman et al., 1998), indicates that the MaPS elicits an accurate portrayal of an individual's food preferences. In addition, the fMRI signals from participants viewing the MaPS are biologically plausible. Images showing highly rewarding food (HF/HS and HF/HCCHO) elicited significantly greater activation in reward relevant regions (such as the insula, putamen, and medial frontal gyrus), compared to images showing low-rewarding food (vegetables). This finding is consistent with previous reports on appetite and reward-related neural systems (Simmons et al., 2005; Beaver et al., 2006; Uher et al., 2006; Cornier et al., 2007; Fuhrer et al., 2008; Schur et al., 2009; Frank et al., 2010). Also, expected regions were more highly activated in response to food images compared to non-food images. The insula and putamen are known to play a central role in the regulation of ingestive behavior beyond primary taste perception, including the memory of the rewarding aspects of eating and integration of information about internal state (Cornier et al., 2009). Brain activation within reward network structures in response to MaPS images appeared to be highly repeatable between the first MaPS viewing and the second. Together, these preliminary findings suggest that the MaPS can provide robust and intuitive stimulation of food reward relevant brain regions during fMRI paradigms in young adults.

The importance of these preliminary findings is that the validity and reliability of food image sets is underreported despite the fact that it could have a major influence on the results of fMRI studies. In particular, image ratings display how participants consciously perceive the images, and it is important that this perception matches that intended by the researcher. Participants perceived images in each MaPS category as intended (high fat foods were viewed as more appetizing, low fat foods were viewed as more nutritious, etc.), and the perceptual properties of the specific images did not distract viewers from the intended perceptions. Not only do subjective participant responses support the validity of the MaPS, but the brain activation in known food-reward pathways also suggests that the food images were perceived as intended. The repeatability of activation in response to the MaPS suggests that the fMRI paradigm could be useful in repeated-measures settings, such as clinical trials in which the goal is to isolate changes in fMRI signals due to treatment rather than to other sources of inter-scan variability. Characterization of food image sets in this fashion provides a rational basis for comparing disparate food image sets and their fMRI results.

Several results may help to inform the design of future food image sets. Although HCCHO foods (flatbreads, rice, plain baked potatoes) are highly palatable, their color is often bland and thus not viewed as visually interesting. HS foods, meanwhile, were highly colorful and engaging. This difference in visual interest could lead to differences in how actively individuals engage with images in the two categories, and thus differences in fMRI signals. Fat content of foods appeared to have an effect on the emotional response to images, possibly due to negative associations of high fat foods (Shepherd and Stockley, 1985; Golay and Bobbioni, 1997; Williams, 2000; Hu et al., 2001; Zaloga et al., 2006; Hoefling and Strack, 2008; Veenstra et al., 2010), leading to another possible source of variability in brain responses. Future food image sets should carefully account for such cognitive or affective factors that could impact the results of fMRI studies with these images.

This study was limited by its homogenous samples of participants and relatively small sample size. Therefore, these results should be considered preliminary. In the behavioral study, all 25 participants were LSU undergraduate students in Psychology, and the majority was female. The 12 fMRI study participants were mostly female Psychology students as well. We did not schedule fMRI scanning sessions around the female participants' menstrual cycles, which may have affected responses to food stimuli. In addition, differences in BMI between the behavioral and fMRI subjects limits the interpretability of the overall findings across the two studies. Replication is needed to ensure the results generalize well to broader populations. In the future, these tests should be conducted on larger, more diverse samples, especially those populations for which food-related imaging studies would be relevant to concurrent behaviors or disease states. In regards to the fMRI paradigm, the block design restricted the ability to dissect responses to individual stimuli. While participants were instructed to begin fasting at 10 p.m. prior to both studies, the length of the fast was not verified. Varying fasting length could have affected our results, and the interpretation of these preliminary findings should be made with caution. Similarly, the overnight fast limits the generalizability of the activation patterns in comparison to more neutral or fed appetitive states, which may be more common in instances when people encounter food stimuli in their natural environment. In addition, the small sample size limited the power to conduct a comprehensive whole-brain analyses, and the more restricted ROI approach may have excluded areas of the brain that are responsive to macronutrient content of food stimuli. Some limitations of the photo set should also be addressed. While the images were well controlled for macronutrient composition, other characteristics relevant to eating behavior (e.g., portion size, food presentation) were less controlled. It would be valuable to include a variety of culturally-relevant and age-appropriate stimuli in future studies with more varied populations. Also, the non-food control images used in the fMRI study were not characterized with the same ratings questions as the food images. Determination of the subjective experience of these images may help to explain fMRI signal differences between views of food and non-food images.

In conclusion, behavioral and fMRI data suggest that ratings of the MaPS images vary predictably and logically with carbohydrate factor and fat content, participant responses to the MaPS images positively correlate to responses on the FPQ, and MaPS images consistently elicit brain activation in appetite and reward relevant brain structures. These preliminary results represent an important step toward rational comparison of food image fMRI studies across labs, as well as multi-site deployment of standardized food image sets.

## Author contributions

JK: Collected behavioral data, oversaw MRI data collection, wrote first draft of paper; SF: Edited manuscript, contributed to data analysis; SR: Analyzed MRI data; PS: Analyzed MRI data; PG: Contributed to data analysis, manuscript writing; CM: Contributed to study design, manuscript writing; KM: Contributed to MRI data collection; JH: Contributed to study design, manuscript writing; FM: Contributed to MRI data collection; JA: Contributed to study design, manuscript writing; OC: Oversaw study and manuscript writing, provided funding.

### Conflict of interest statement

The authors declare that the research was conducted in the absence of any commercial or financial relationships that could be construed as a potential conflict of interest.
