# Supplementary material for: Perceptual Characterization of the Macronutrient Picture System (MaPS) for Food Image fMRI
Source: Front Psychol. 2018 Jan 26;9:17. doi: 10.3389/fpsyg.2018.00017 (PMC5790788; doi:10.3389/fpsyg.2018.00017)

**Appendix A:** Example of MaPS Images

|  | High Sugar (HS) | High Complex Carbohydrate (HCCHO) | Low Carbohydrate/High Protein (LCHO/HP) |
| --- | --- | --- | --- |
| High Fat  (HF) | 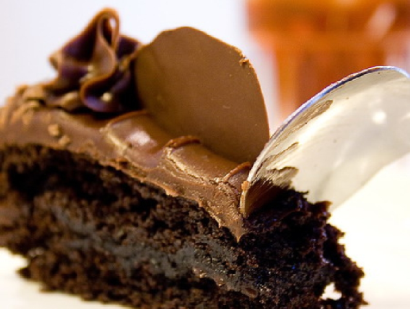 | 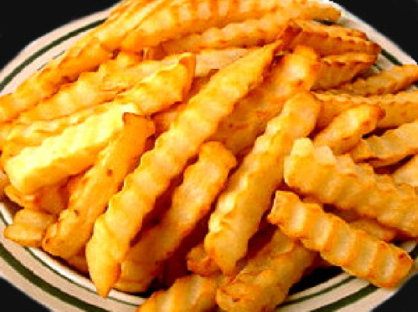 | 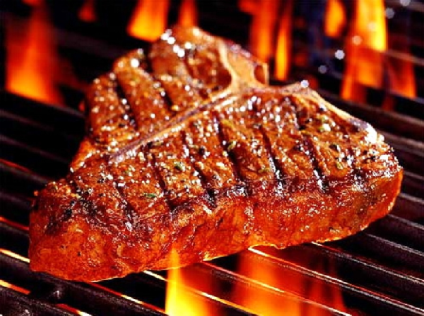 |
| Low Fat  (LF) | 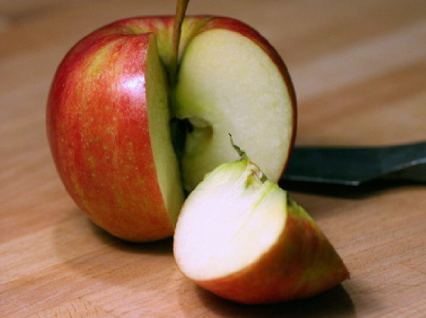 | 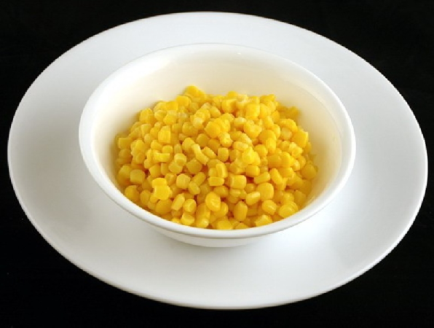 | 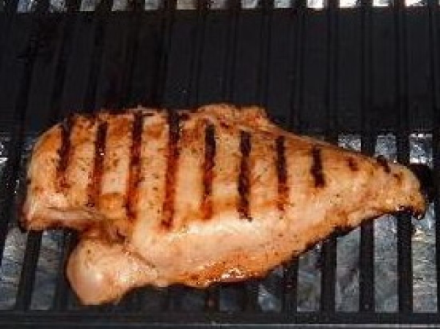 |


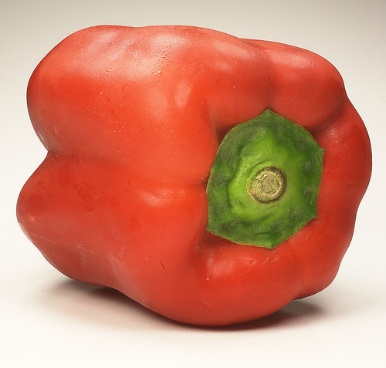
Example Vegetable Images


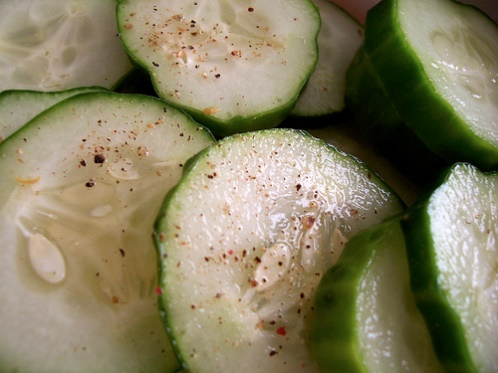

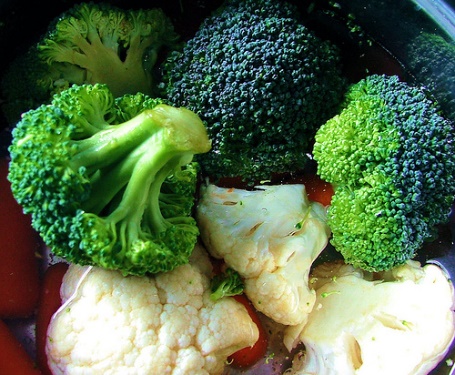


Example Control Images


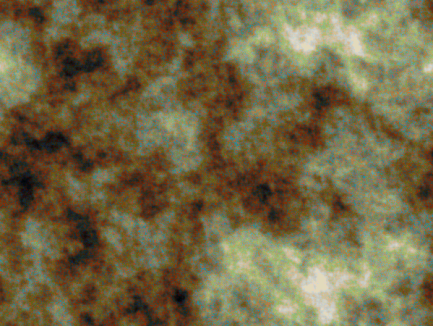

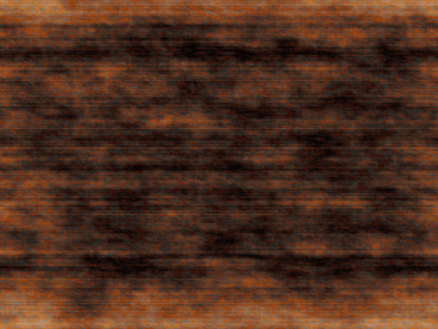

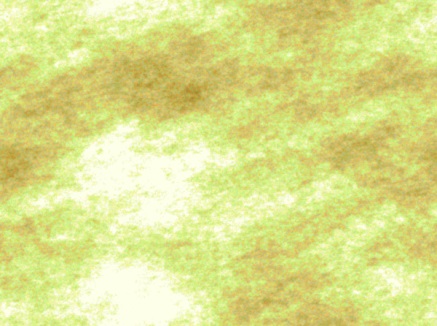

Supplement: Supplementary file 1 [file AppendixA.DOCX]
