# Supplementary material for: Perceptual Characterization of the Macronutrient Picture System (MaPS) for Food Image fMRI
Source: Front Psychol. 2018 Jan 26;9:17. doi: 10.3389/fpsyg.2018.00017 (PMC5790788; doi:10.3389/fpsyg.2018.00017)
Supplement: Supplementary file 2 [file AppendixB.DOCX]

**Appendix B:** Subjective Ratings

Average Image Ratings

|  | HF/HS | LF/HS | HF/HCCHO | LF/HCCHO | HF/LCHO/HP | LF/LCHO/HP |
| --- | --- | --- | --- | --- | --- | --- |
| Interest | 5.64 ± 1.63 | 5.15 ± 1.87 | 5.29 ± 1.31 | 4.04 ± 1.69 | 4.83 ± 1.18 | 4.47 ± 1.23 |
| Appetite | 5.68 ± 2.00 | 4.71 ± 1.87 | 6.23 ± 1.19 | 4.79 ± 1.67 | 5.32 ± 1.91 | 4.70 ± 1.61 |
| Nutrition | 0.83 ± 0.68 | 4.47 ± 0.56 | 1.49 ± 0.75 | 4.53 ± 1.23 | 4.53 ± 1.31 | 6.81 ± 1.26 |
| Emotion | 3.78 ± 1.93 | 3.49 ± 2.06 | 3.67 ± 1.81 | 2.86 ± 1.63 | 3.39 ± 1.75 | 3.16 ± 1.87 |
| Liking | 5.36 ± 1.80 | 4.84 ± 1.62 | 6.06 ± 1.19 | 5.01 ± 1.36 | 5.28 ± 1.61 | 4.84 ± 1.32 |
| Frequency | 2.35 ± 0.89 | 2.77 ± 1.08 | 3.68 ± 0.97 | 3.23 ± 1.10 | 3.37 ± 1.20 | 3.62 ± 1.32 |

Average FPQ and MaPS Liking Ratings

|  | (HF/HS) | (LF/HS) | (HF/HCCHO) | (LF/HCCHO) | (HF/LCHO/HP) | (LF/LCHO/HP) |
| --- | --- | --- | --- | --- | --- | --- |
| FPQ | 5.69±1.78 | 5.61±1.72 | 6.16±1.25 | 5.38±1.36 | 6.06±1.51 | 5.36±1.32 |
| MaPS  Image Liking | 5.36±1.80 | 4.83±1.61 | 6.06±1.19 | 5.01±1.36 | 5.28±1.61 | 4.84±1.32 |
