# Supplementary material for: Perceptual Characterization of the Macronutrient Picture System (MaPS) for Food Image fMRI
Source: Front Psychol. 2018 Jan 26;9:17. doi: 10.3389/fpsyg.2018.00017 (PMC5790788; doi:10.3389/fpsyg.2018.00017)
Supplement: Supplementary file 3 [file AppendixC.DOCX]

**Appendix C:** Regions of Interest (ROIs) in fMRI study


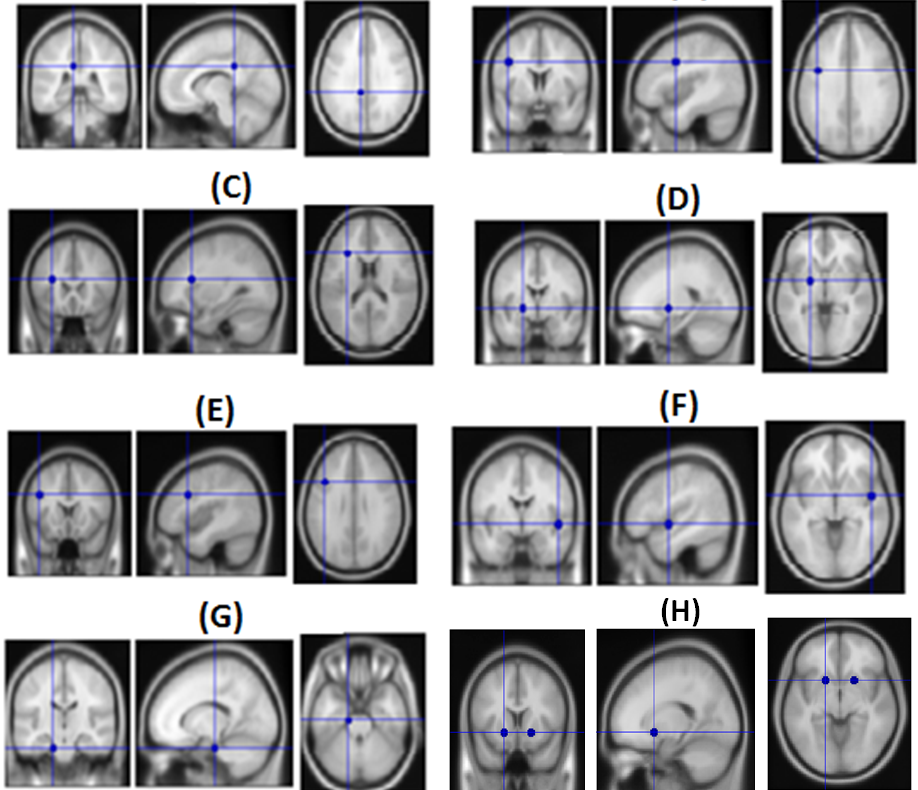


Regions of Interest (shown with blue cross-hairs) include the cingulate (A) and precentral gyri (B), insula (C), putamen (D), medial frontal (E), superior temporal (F), parahippocampal gyri (G) and ventral striatum (H).

MNI Coordinates of ROIs

| (A) | Cingulate gyrus | x = -9 , y = -37, z = 31 |
| --- | --- | --- |
| (B) | Precentral gyrus | x = -42, y = 4, z = 33 |
| (C) | Insula | x = -30, y = 22, z = 18 |
| (D) | Putamen | x = -21, y = -2, z = -2 |
| (E) | Medial Frontal gyrus | x = -42, y = 16, z = 29 |
| (F) | Superior Temporal gyrus | x = 48 , y = -3, z = -4 |
| (G) | Parahippocampal gyrus | x = -15, y = -19, z = 27 |
| (H) | Ventral Striatum | x =±18, y = 12, z = −6 |
